# Transcriptional study of hyperoxaluria and calcium oxalate nephrolithiasis in male rats: Inflammatory changes are mainly associated with crystal deposition

**DOI:** 10.1371/journal.pone.0185009

**Published:** 2017-11-01

**Authors:** Sunil Joshi, Wei Wang, Saeed R. Khan

**Affiliations:** 1 Department of Pathology, Immunology & Laboratory Medicine, College of Medicine, University of Florida, Gainesville, Florida, United States of America; 2 Department of Urology, College of Medicine, University of Florida, Gainesville, Florida, United States of America; The University of Manchester, UNITED KINGDOM

## Abstract

Hyperoxaluria associated with renal deposition of calcium oxalate (CaOx) crystals causes renal injury and inflammation leading to number of diseases including chronic kidney disease (CKD). It is however, not been possible to separate the renal consequences of hyperoxaluria from that of CaOx crystal deposition. We decided to utilize ethylene glycol (EG) model where hyperoxaluria and CaOx crystal deposition can be separated in time. To test our hypothesis, male rats were made hyperoxaluric by administering EG, rats were euthanized and kidneys were extracted on day 14, when occasional crystal is seen in the kidneys and day 28, when all animals have developed renal CaOx crystal deposits. Total RNA was extracted for microarray analysis and genome wide analysis of differentially expressed genes was performed to investigate differences between hyperoxaluria and crystal induced alterations in the kidneys. Immunohistochemical and Hematoxylin and Eosin (H&E) staining was also done for macromolecules with significant role in stone formation. All EG fed rats became hyperoxaluric by day 7, showed a few crystal deposits on day 14, and had heavy crystal deposition by day 28. There were significant changes in the expression of genes encoding for NADPH Oxidases; macromolecular crystallization modulators; genes involved in inflammasome activation; and osteogenic marker genes. Results demonstrate major differences between hyperoxaluria and CaOx crystal induced changes in the kidneys. Injury and inflammation are mainly associated with crystal deposition indicating significant role played by crystal retention.

## Introduction

Oxalate is an organic compound found naturally in many foods such as spinach, rhubarb, beets, wheat bran, strawberries, almonds and peanuts [1]. Higher concentration of oxalate in the human body can lead to a number of pathological conditions including hyperoxaluria which can further lead to a large spectrum of diseases such as nephrocalcinosis, cardiomyopathy, cardiac conductance disorders, systemic oxalosis, renal failure and specially CaOx kidney stones. Hyperoxaluria is excessive urinary excretion of oxalate and can be classified into different types such as primary, secondary, idiopathic and enteric hyperoxaluria caused by a variety of factors including genetic defects or mutation of specific genes (Primary hyperoxaluria), eating oxalate rich foods (Secondary hyperoxaluria), interplay of dietary, genetic and environmental factors with unknown causes (Idiopathic hyperoxaluria) and due to fat malabsorption, jejunal bypass surgery, and modern gastric bypass (Enteric hyperoxaluria) [2–6]. Kidney stone formation is a chronic disease which is becoming widespread nowadays, both in the United States and globally. Due to increased prevalence in the US, it is causing a significant economic burden on US health care [7]. According to National Health and Nutrition Examination Surveys (NHANES) of US adults, kidney stone prevalence increased from 5.2% (1988–1994) to 8.4% (2007–2010) [7] and a recent study showed that the number of kidney stone patients increased from 1 in 20 persons to 1 in 11 persons since 1994. Among men, the prevalence was 10.6% as compared to 7.1% among women and Caucasians were more likely to report a history of kidney stones as compared to African American and Hispanic individuals [8]. Also, recent studies show a link between stone formation and hypertension, chronic kidney disease and even end stage renal disease [9–12].

To understand the pathogenesis of hyperoxaluria and CaOx stone formation, many animal models have been developed [13–15]. Two rat models in which hyperoxaluria is induced by the administration of the ethylene glycol (EG) or hydroxy-L-proline (HLP) [16] have been studied in detail. Previous research has shown that hyperoxaluria and renal CaOx crystal deposition produced reactive oxygen species (ROS), upregulated mineralization modulators [17–20], caused lipid peroxidation, and renal cellular injury [16,21–23]. The free radicals or ROS appeared to play significant role in the expression of various factors and pathways [24,25], involved in the activation of inflammasomes [23], production of macromolecules implicated in crystal formation and retention [26,27], and osteogenesis [28]. These studies were however performed in the kidneys with extensive CaOx crystal deposition. As a result, nature of instigation, hyperoxaluria or CaOx crystals could not be established. Current study was performed using EG as hyperoxaluria inducing agent where, in our laboratory, administration of 0.75% EG in drinking water to male Sprague-Dawley rats lead to consistent hyperoxaluria by 2 weeks and CaOx crystal deposition by 4 weeks. We analyzed changes in global transcriptome of renal tissue, following development of hyperoxaluria at 2 weeks and CaOx crystal deposition or nephrolithiasis at 4 weeks of EG treatment. Emphasis was placed on the genes and pathways involved in production of reactive oxygen species, crystallization modulators, development of inflammation and osteogenesis. Results presented herein demonstrate that several pathways were commonly expressed at both day 14 and 28. Similarly, many significantly differentially expressed genes were common between day 14 and day 28. However significant differences were found between day 14 and 28, in the relative expression of genes involved in osteogenesis, activation of inflammasome and macromolecular production.

## Materials and methods

### Animal model

Eight weeks old, male Sprague- Dawley rats (n = 40), with average weight of about 110–120 grams, were bought from Harlan Laboratories (Harlan, Tampa, Florida). The rats were accustomed for 2 weeks within the Animal care facilities in their normal as well as metabolic cages for 2 days within the 2-week period at University of Florida before any experimental procedures and prior to start their dietary regimen. All the cages, food and water were sterilized by autoclaving before doing any experiments. Rats were divided into 2 groups (n = 20): Group 1 rats (control) were fed normal rat chow and sterile water and divided into 4 subgroups (day7, day14, day21 and day28) of 5 rats in each subgroup, Group 2 rats had a similar diet to Group 1 rats except were fed 1.25% Ethylene glycol (EG) in drinking water. Group 2 rats were also divided into 4 subgroups (day7, day14, day21, and day 28) of 5 rats in each subgroup. The rats were placed in metabolic cages for urine collection 1 day before sacrificing. On day 7, 5 rats from each group (total 10) belonging to day7 subgroup were euthanized and the kidneys freshly extracted. Rats were anesthetized with an injection of Pentobarbital IP. Once the rats were anesthetized and reached a surgical plane of anesthesia demonstrated by the lack of a pedal withdrawal reflex, their body cavity was opened to expose their internal organs. The rats were perfused with sterile saline and heparin was flushed throughout the body. Shortly after starting the perfusion process an over dose of Pentobarbital was added to ensure that the animal was dead. This study was carried out in strict accordance with the recommendations in the Guide for the Care and Use of Laboratory Animals of the National Institute of Health. All research was approved by the Institutional Animal Care and Use Committee (IACUC) at University of Florida (IACUC study # 201101850).

One Kidney was carefully divided into cortex and medulla and was stored in RNA later solution at -20 degrees for RNA isolation and at -80 degrees for protein isolation. The other kidney was cut into parts and was also kept in formalin to be embedded in paraffin for histological analysis. Similar procedure was followed for day14, 21, and day 28.

### Urine collection and analysis

Every week, 24-hour urine was collected with 0.02% sodium azide to prevent bacterial growth. Rats were placed in metabolic cages for collection of urine. After determining volume and pH, urine was aliquoted for various assays. Light microscopy was used to determine crystalluria. Urinary lactate dehydrogenase (LDH) was measured using CytoTox® Non-Radioactive Cytotoxicity Assay (Cat. # G1780) as per the manufacturer’s instructions (Promega Corporation, Madison, WI). Urinary creatinine was measured using the Colorimetric Microplate Assay for Creatinine (Product No. CR 01) from Oxford Biomedical Research, Oxford, MI as per their protocol. Urinary Oxalate was measured using the Oxalate assay kit (Cat. # 591D) from Trinity Biotech, Bray CO as per the manufacturer’s instructions, similar to methods described in our previous studies [29]. The data was further analyzed using 2-way Analysis of Variance in Graph Pad Prism V 5.0 (La Jolla, CA).

## RNA extraction and differential expression of genes by microarray analysis

The total RNA from each of the different specimens was isolated from the kidneys of rats within the two different treatment groups simultaneously using the RNeasy Mini-Kit (QIAGEN, Valencia, CA), as per the manufacturer’s instructions as described in our previous studies [23,30,31].

## Microarray analysis and data mining

The microarray analysis was done in the Interdisciplinary Center for Biotechnology Research (ICBR), University of Florida, using the Agilent 8x60k single color arrays under the Agilent microarray incentive program. Under this program, we used 2 different time points as day 14 and day 28, with the control, and EG treated group. There were four replications for all the samples.

The data analysis of these samples was done at the Interdisciplinary Center for Biotechnology Research (ICBR) using the Bioconductor limma (Linear models for microarray analysis) package using R [32]. Before the analysis, the individual signal intensity values retrieved from the microarray probes were log transformed (using 2 as a base) and normalization was done for all the individual samples within all the groups in this study. After normalizing the signal intensity values for each of the arrays, the Student’s t-test was used to do a probe-by probe comparison between two groups concurrently. For each comparison, the fold change (FC) and p-value was calculated for each gene based on the n = 4 replicate samples within each experimental group and heat map, raw data box plot, relative log expression (RLE), and volcano plots were drawn for each comparison i.e. (Group2-1). The heat map was drawn using heat map package in R [32] and gene rows were clustered according to the fold change levels.

GO:TERM and KEGG pathway analyses based on differentially-expressed genes were carried out using DAVID (Database for Annotation, Visualization of Integrated Discovery) enrichment analysis tool from National Institute of Allergy and Infectious Diseases (NIAID), NIH [33]. The DAVID knowledgebase is built around DAVID gene concept and is intended to facilitate high throughput gene functional analysis. Cluster analyses of genes permitted identification of biological processes, cellular component, and molecular function ontology. All Microarray data have been deposited with Gene Expression Omnibus (GSE-89028).

### Histological and immunohistological examinations

Kidneys from all the rats were extracted after euthanasia, one of which was used for RNA preparation, while the second was placed in 10% phosphate buffered formalin for 24 hours for histological analysis as previously described [31]. The tissue already fixed with formalin were embedded in paraffin and cut into sections of 5μm thickness. Deparaffinization of paraffin-embedded slides was performed by xylene immersion and subsequent dehydration in ethanol. Kidney sections were processed for immunohistochemistry using specific primary antibodies reactive to Osteopontin (OPN- Polyclonal Rb-Anti-OPN, 1:100 dilute, Abcam Cat. # ab8448), Matrix Gla-Protein (MGP- Polyclonal Rb-Anti-MGP, 1:50 dilute, Santa Cruz Cat. # 66965), Kidney Injury Molecule (KIM-1 Polyclonal Rb-Anti-KIM1, 1:100 dilute, Abcam Cat. # ab47635), Collagen (Col1a1 Polyclonal Rb-Anti-Col1a1, 1:00 dilute, Novus Biologicals Cat. # NB600-408), Fibronectin 1 (Fn 1 Monoclonal Ms-Anti-FN1, 1:50 dilute, Santa Cruz Cat. # sc59826), Tamm-Horsfall Protein (THP Polyclonal Rb-Anti-THP, 1:50 dilute, Santa Cruz Cat. # sc20631), Fetuin B (Fetub Monoclonal Rb-Anti-Fetuin B, 1:50 dilute, Abcam Cat. # ab191569) NADPH oxidase 2 (NOX2 Polyclonal Rb-Anti-Nox2, 1:100 dilute, (Abcam Cat. # ab31092) and NADPH oxidase 4 (NOX4 Polyclonal Rb-Anti-Nox4, Abcam Cat. # ab61248). Isotype controls were performed using rabbit IgG. Slides were incubated for 30 min in biotinylated goat anti-rabbit IgG followed by incubation with biotinylated horseradish peroxidase using the Vectastain® ABC kit. Staining was developed by addition of diaminobenzidine (DAB) substrate (Vector Labs, Burlingame, CA) and counterstained with hematoxylin. To ensure that positive infiltrate staining for these antibodies was not due to high background staining, an additional run was performed using 10 mM citrate buffer for antigen-retrieval with all other procedures unchanged. Antigen-retrieval was carried out in 25 mMTris/EDTA buffers, pH 9.1 at 60°C for 20 min under 18 psi pressure. Images were taken using the Zeiss Axiovert 200M microscope (Carl Zeiss Microimaging, Inc., Thornwood, NY).

## Results

### Histology and urinary assays

**Fig 1:**
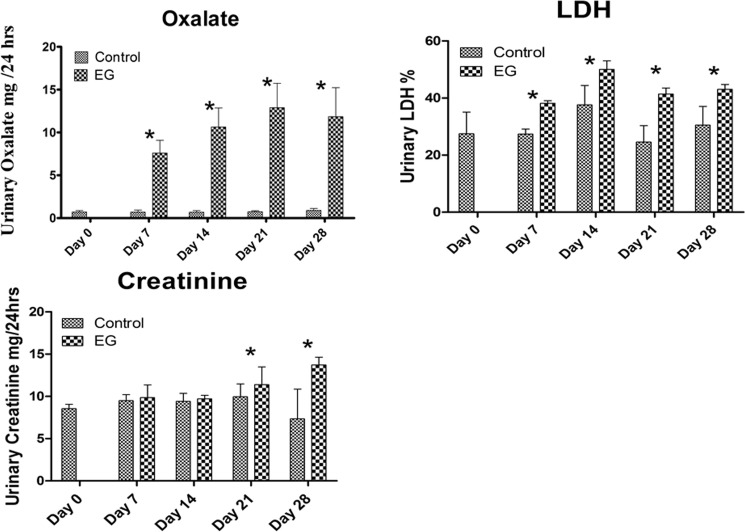
Urinary excretion of control and ethylene glycol (EG) treated rats. A) EG fed rats showed significant increase in urinary oxalate (mg/24hrs) excretion on day 7, 14, 21 and 28 respectively B) Urinary Lactate Dehydrogenase (LDH) was elevated in the EG fed rats on Day 7, 14, 21 and 28 respectively as compared to the control rats, Cytotoxicity level of LDH was measured as expressed as percentage (%) and C) Urinary Creatinine (mg/24hrs) was significantly higher on day 21 and 28 in the EG fed rats as compared to the control group. The data was analyzed using Graph Pad Prism with 2-way Analysis of Variance. *P < 0.05 Control versus EG within the same time period.

As anticipated, EG administration to the rats produced hyperoxaluria and CaOx nephrolithiasis i.e. crystal deposition in the kidneys. There was a significant increase in urinary excretion of oxalate by the rats consuming EG as compared to the control group peaking at day 14 and remaining significantly high till day 28. Urinary LDH increased significantly for Days 7, 14, 21and 28 for the EG consuming rats as compared to the control rats **(Fig 1)**. Urinary creatinine increased significantly on day 21 and day 28 in the EG fed rats as compared to the control group **(Fig 1)**.

**Fig 2:**
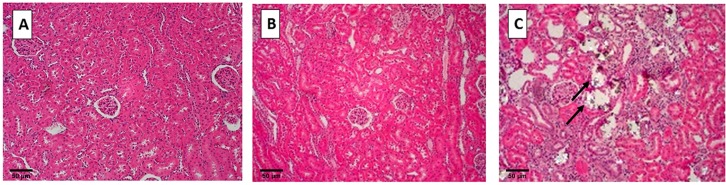
Hematoxylin and eosin staining of paraffin embedded kidneys examined under polarized light. A) Control rats, no crystal deposition and B) rats fed 1.25% Ethylene Glycol (EG), observed on Day 14 (Hyperoxaluric stage) and C) rats fed 1.25% EG with lots of birefringent crystals observed on Day 28 (Nephrolithiasis stage), shown by black arrows Magnification 10x, Scale bar 50μm.

When sacrificed on day 28, kidneys of control rats appeared normal without any crystal deposits. Kidneys of rats sacrificed on day 14 of EG treatment also did not contain easily detectable CaOx crystal deposits. However, kidneys of rats sacrificed on day 28 of the EG administration showed copious amounts of birefringent CaOx crystals in the kidneys (**Fig 2**). Most of the crystals were found in the cortex and outer medulla, with few on the inner medulla. Crystals were generally located in the tubular lumens of the distal tubules and collecting ducts. Most of the renal tubules where crystals were present were distended. Crystal deposition also caused tissue disruption.

### Microarray analysis and data mining

Of more than 30,000 rat genes represented on the arrays, 938 and 2400 genes were found to be significantly differentially expressed and also up regulated in the renal tissue, respectively for Day 14 and Day 28 (Log FC ≥ 0.3), in comparison between the control and the EG-fed rats. There were 440 (Day 14) and 1902 (Day28) genes that were significantly differentially expressed and highly up regulated.

Using Kyoto Encyclopedia of Genes and Genome Pathway analysis (KEGG) to curate the differentially-expressed genes, several gene clusters were identified that defined specific biological processes for the EG-fed rats, on Day 14 and Day 28 respectively as presented in **Tables 1 and 2.**

**Table 1:** Functional annotation charts showing differentially expressed pathways on Day 14 of highly expressed genes in the EG group.

Category	Signaling pathway	Gene count	Percentage	p-value	Benjamini
KEGG-PATHWAY	Complement and coagulation cascades	20	2.6	5.3E-10	7.8E-8
KEGG-PATHWAY	ECM-receptor interaction	18	2.3	2.8E-7	2.0E-5
KEGG-PATHWAY	Focal adhesion	25	3.2	2.6E-5	1.3E-3
KEGG-PATHWAY	Leucocyte transendothelial migration	18	2.3	4.1E-5	1.5E-3
KEGG-PATHWAY	Cell adhesion molecules (CAMS)	20	2.6	1.1E-4	3.1E-3
KEGG-PATHWAY	Systemic lupus erythematosus	13	1.7	1.4E-3	3.5E-2
KEGG-PATHWAY	Chemokine signaling pathway	18	2.3	4.3E-3	8.8E-2
KEGG-PATHWAY	Natural killer cell mediated cytotoxicity	12	1.6	9.8E-3	1.7E-1
KEGG-PATHWAY	Prion diseases	6	0.8	2.7E-2	3.7E-1
KEGG-PATHWAY	Hematopoietic cell lineage	9	1.2	3.7E-2	4.3E-1
KEGG-PATHWAY	Pathways in cancer	24	3.1	3.9E-2	4.1E-1
KEGG-PATHWAY	Tight junction	12	1.6	5.5E-2	5.0E-1
KEGG-PATHWAY	Cytokine-cytokine receptor interaction	16	2.1	6.2E-2	5.1E-1
KEGG-PATHWAY	Viral myocarditis	9	1.2	6.4E-2	5.0E-1
KEGG-PATHWAY	Fc gamma R-mediated phagocytosis	9	1.2	6.8E-2	5.0E-1
KEGG-PATHWAY	Cell cycle	11	1.4	9.2E-2	5.9E-1
KEGG-PATHWAY	Glutathione metabolism	6	0.8	9.9E-2	6.0E-1

The gene heading indicates number of genes mapped to an ontology category. P-values derived from Fisher’s exact test and Benjamini multiple test correlation. Gene ontology (GO) analysis and pathway analysis of genes also done using DAVID (Database for Annotation, Visualization of Integrated Discovery) enrichment analysis tool from National Institute of Allergy and Infectious diseases (NIAID), NIH.

**Table 2:** Functional annotation charts showing differentially expressed pathways of highly expressed genes in the EG group on Day 28.

Category	Signaling pathway	Gene count	Percentage		p-value		Benjamini
KEGG-PATHWAY	Hematopoietic cell lineage	24	1.7		1.0E-7		1.7E-5
KEGG-PATHWAY	ECM-receptor interaction	23	1.6		9.5E-7		7.9E-5
KEGG-PATHWAY	Cell cycle	29	2.0		2.7E-6		1.5E-4
KEGG-PATHWAY	DNA replication	14	1.0		3.7E-6		1.6E-4
KEGG-PATHWAY	Complement and coagulation cascades	19	1.3		2.1E-5		7.1E-4
KEGG-PATHWAY	Cell adhesion molecules (CAMS)	30	2.1		2.5E-5		6.9E-4
KEGG-PATHWAY	Chemokine signaling pathway	31	2.2		1.5E-4		3.7E-3
KEGG-PATHWAY	Cytokine-cytokine receptor interaction	34	2.4		1.8E-4		3.8E-3
KEGG-PATHWAY	p53 signaling pathway	16	1.1		4.5E-4		8.3E-3
KEGG-PATHWAY	Ribosome	18	1.2		7.0E-4		1.2E-2
KEGG-PATHWAY	Focal adhesion	32	2.2		7.2E-4		1.1E-2
KEGG-PATHWAY	NOD-like receptor signaling pathway	15	1.0		7.4E-2		1.0E-1
KEGG-PATHWAY	Primary immunodeficiency	10	0.7		2.0E-3		2.6E-2
KEGG-PATHWAY	Leucocyte transendothelial migration	20	1.4		4.9E-3		5.7E-2
KEGG-PATHWAY	Natural killer cell mediated cytotoxicity	18	1.2		5.6E-3		6.1E-2
KEGG-PATHWAY	Systemic lupus erythematosus	16	1.1		1.1E-2		1.1E-1
KEGG-PATHWAY	Hypertrophic cardiomyopathy (HCM)	15	1.0		1.4E-2		1.3E-1
KEGG-PATHWAY	Pathways in cancer	40	2.8		1.7E-2		1.5E-1
KEGG-PATHWAY	Gap junction	14	1.0		2.3E-2		1.8E-1
KEGG-PATHWAY	Dilated cardiomyopathy	15	1.0		2.4E-2		1.8E-1
KEGG-PATHWAY	Glutathione metabolism	10	0.7		2.8E-2		2.0E-1
KEGG-PATHWAY	Terpenoid backbone biosynthesis	5	0.3		2.9E-2		2.0E-1
KEGG-PATHWAY	Graft-versus-host disease	10	0.7		3.1E-2		2.1E-1
KEGG-PATHWAY	Toll-like receptor signaling pathway	14	1.0		4.9E-2		3.0E-1
KEGG-PATHWAY	Small cell lung cancer	13	0.9		5.7E-2		3.2E-1
KEGG-PATHWAY	Apoptosis	13	0.9		6.6E-2		3.6E-1
KEGG-PATHWAY	Viral myocarditis	13	0.9		7.6E-2		3.9E-1
KEGG-PATHWAY	Type I diabetes mellitus	10	0.7		7.6E-2		3.8E-1
KEGG-PATHWAY	Steroid hormone biosynthesis	8	0.6		7.8E-2		3.7E-1
KEGG-PATHWAY	Fc gamma R-mediated phagocytosis	13	0.9		8.1E-2		3.8E-1
KEGG-PATHWAY	Drug metabolism	8	0.6		8.6E-2		3.9E-1
KEGG-PATHWAY	Renin-angiotensin system	5	0.3		9.2E-2		4.0E-1
KEGG-PATHWAY	T cell receptor signaling pathway	15	1.0		9.3E-2		3.9E-1

The gene heading indicates number of genes mapped to an ontology category. P-values derived from Fisher’s exact test and Benjamini multiple test correlation.

Interestingly, the differentially-expressed genes identifying the various KEGG signaling pathways revealed that, there were 17 and 33 pathways that were significant on Day 14 and Day 28 respectively. Of these, 15 pathways were common between Day 14 and Day 28 **(Table 3)**.

**Table 3:** Common pathways between Day 14 and Day 28 of highly expressed genes in the EG group.

KEGG #	Signaling Pathway	Day 14		Day 28	
		Gene Count	Percentage	Gene Count	Percentage
1	Hematopoietic cell lineage	9	1.2	24	1.7
2	ECM-receptor interaction	18	2.3	23	1.6
3	Cell cycle	11	1.4	29	2
4	Complement and coagulation cascades	20	2.6	19	1.3
5	Cell adhesion molecules (CAMs)	20	2.6	30	2.1
6	Chemokine signaling pathway	18	2.3	31	2.2
7	Cytokine-cytokine receptor interaction	16	2.1	34	2.4
8	Focal adhesion	25	3.2	32	2.2
9	Leukocyte transendothelial migration	18	2.3	20	1.4
10	Natural killer cell mediated cytotoxicity	12	1.6	18	1.2
11	Systemic lupus erythematosus	13	1.7	16	1.1
12	Pathways in cancer	24	3.1	40	2.8
13	Glutathione metabolism	6	0.8	10	0.7
14	Viral myocarditis	9	1.2	13	0.9
15	Fc gamma R-mediated phagocytosis	9	1.2	13	0.9

Gene ontology (GO) analysis and pathway analysis of genes also done using DAVID (Database for Annotation, Visualization of Integrated Discovery) enrichment analysis tool from National Institute of Allergy and Infectious diseases (NIAID), NIH

We also found that there were 18 unique pathways for the Day 28 (Nephrolithiasis) group **(Table 4)** and 2 pathways that were unique for Day 14 (Hyperoxaluria) group **(Table 5)** when we compared the pathways.

**Table 4:** Signaling pathways that are unique to day 28 with heavy CaOx crystal deposition (Nephrolithiasis) when compared with day 14 (Hyperoxaluric condition).

KEGG Pathway	Signaling pathway	Gene Count	Percentage
1	DNA replication	14	1.0
2	P53 signaling pathway	16	1.1
3	Ribosome	18	1.2
4	Nod–like receptor signaling pathway	15	1.0
5	Primary immunodeficiency	10	0.7
6	Hypertrophic cardiomyopathy	15	1.0
7	Gap junction	14	1.0
8	Dilated cardiomyopathy	15	1.0
9	Terpenoid backbone biosynthesis	5	0.3
10	Graft-versus-host disease	10	0.7
11	Toll-like receptor signaling pathway	14	1.0
12	Small cell lung cancer	13	0.9
13	Apoptosis	13	0.9
14	Type-1 diabetes mellitus	10	0.7
15	Steroid hormone biosynthesis	8	0.6
16	Drug metabolism	8	0.6
17	Renin-angiotensin system	5	0.3
18	T-cell receptor signaling pathway	15	1.0

**Table 5:** Signaling pathways that were unique to day 14 (Hyperoxaluria) only when compared with day 28.

KEGG Pathway	Signaling pathway	Gene Count	Percentage
1	Prion disease	6	0.8
2	Tight junction	12	1.6

Major pathways that were unique to nephrolithiasis group and were highly significantly expressed included P53 signaling, Nod-like receptor signaling, hypertrophic cardiomyopathy, toll like receptor signaling, and T cell receptor signaling. The two unique pathways for hyperoxaluric group were the prion disease and tight junction signaling.

**Fig 3:**
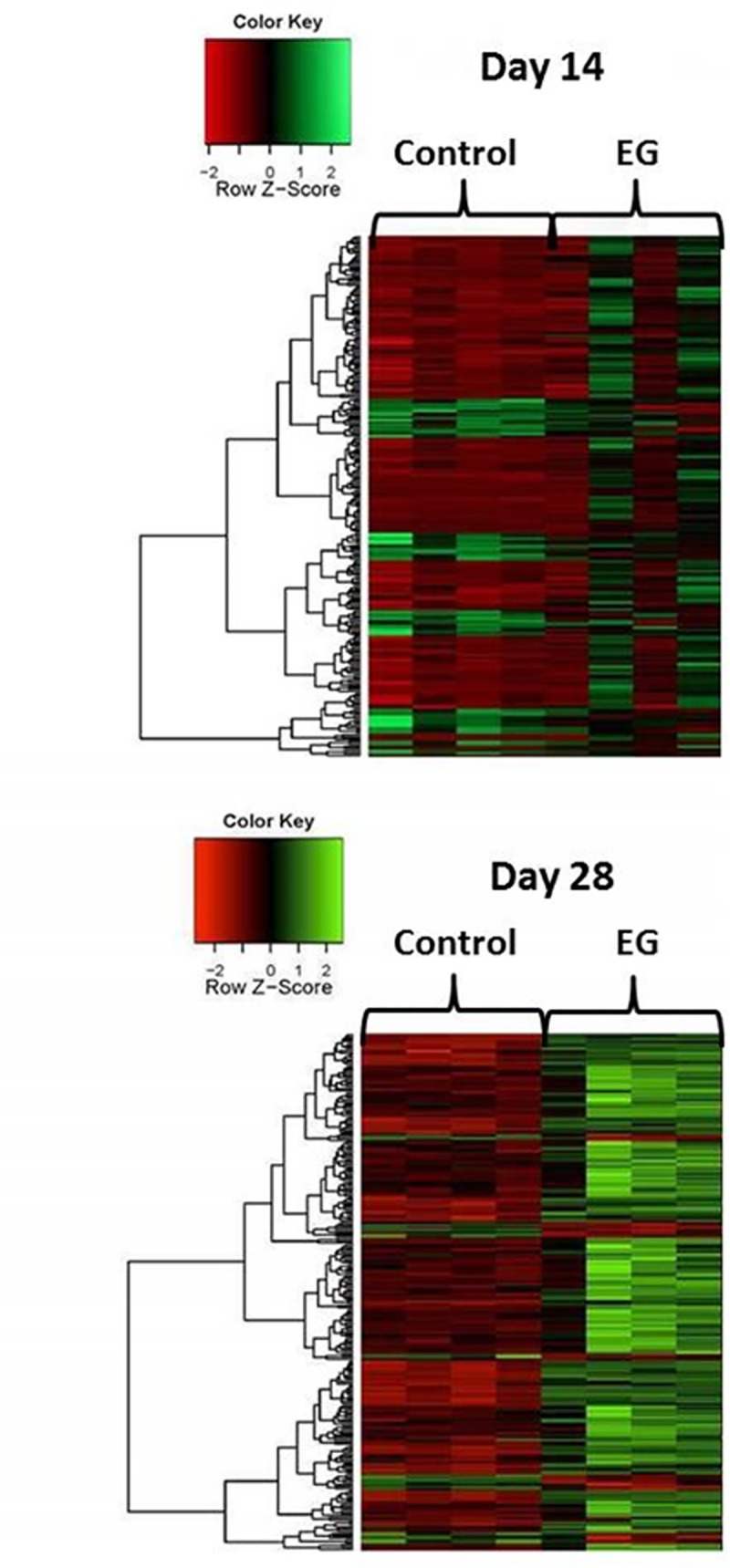
Heat map showing up as well as down regulated genes. Top map compares 4 control rats to 4 rats after EG treatment on day 14 and bottom map on Day 28. Significant differences are apparent between control and EG treated rats on both days as well as between the treated rats on day 4 when rats were mostly hyperoxaluric and crystals free vs day 28 with heavy CaOx crystals deposits in the kidneys.

**Fig 3** shows microarray data as heat maps. It shows up and down regulated genes on day 14 and 28 after EG treatment compared to their controls. Significant differences are apparent between controls and EG treated rats as well as between 14 day and 28 days of treatments.

Based upon previous results and our earlier investigations with HLP fed rats [31], we looked for genes encoding for NOX 4, NOX 2, p22phox, GPX2 as well as KIM-1 **(Table 6)**. They were all significantly upregulated in rats given EG. Expression of NOX 4 and NOX 2 was however higher on day 14 than on day 28. Expression of p22Pphox and Kim-1 was on the other hand higher on day 28 than on day 14.

**Table 6:** Relative expression of important genes in ethylene glycol (EG) fed rat vs control group, along with their p-values derived from microarray analysis and data mining.

GENE ID	DESCRIPTION	DAY 14		DAY 28	
		Log Fold Change	p-value	Log Fold Change	p-value
**NOX4**	NADPH Oxidase 4 / Renal NADPH Oxidase	0.578	0.037	0.387	0.035
**NOX2/CYBB**	NADPH Oxidase 2/ Cytochrome B-245 Beta Chain	0.952	0.626	0.518	0.840
**P22/CYBA**	P22Phox / Cytochrome B-245 Alpha Chain	0.202	0.268	0.290	0.080
**GPX2**	Glutathione Peroxidase 2	1.583	0.028	2.742	0.0001
**KIM1**	Kidney Injury Molecule 1	1.870	0.065	4.390	0.0001
**OPN/SPP1**	Osteopontin / Secreted Phosphoprotein 1	2.400	0.022	4.160	0.00002
**MCP1**	Monocyte Chemoattractant Protein 1	0.175	0.638	1.740	0.201
**MGP**	Matrix Gla Protein	0.705	0.108	0.807	0.0019
**FETUB**	Fetuin B	0.285	0.643	0.420	0.053
**THP/UMOD**	Tamm-Horsfall Protein / Uromodulin	-0.246	0.442	-0.426	0.063
**LCN2**	Lipocalin 2	1.807	0.058	2.178	0.004
**FN1**	Fibronectin 1	0.078	0.393	1.095	0.047
**CLU**	Clusterin	1.773	0.041	2.086	0.0003
**CD44**	Cell Surface glycoprotein CD44	0.626	0.137	1.586	0.00001
**AMBP**	Alpha-1- Microglobulin / Bikunin Precursor	0.210	0.020	0.258	0.020
**PYCARD**	PYD And CARD Domain-Containing Protein	0.422	0.096	0.393	0.097
**NLRP3**	NLR Family Pyrin Domain Containing 3	0.024	0.807	0.090	0.903
**IL-18**	Interleukin 18	0.076	0.562	0.092	0.406
**TXNIP**	Thioredoxin Interacting Protein	0.183	0.448	0.130	0.498
**IL-1β**	Interleukin 1 Beta	0.194	0.345	0.799	0.007
**CASP1**	Caspase 1	-0.0041	0.979	0.371	0.010
**RUNX1**	Runt Related Transcription Factor 1	0.557	0.136	1.063	0.00007
**RUNX2**	Runt Related Transcription Factor 2	0.042	0.794	0.099	0.372
**KRT18**	Keratin 18 / Cytokeratin 18	0.447	0.077	0.706	0.009
**KRT8**	Keratin 8 / Cytokeratin 8	0.213	0.170	0.238	0.104
**VIM**	Vimentin	0.237	0.405	0.412	0.008
**COL1A1**	Collagen Type 1 Alpha 1 Chain	0.474	0.235	0.591	0.009
**COL1A2**	Collagen Type 1 Alpha 2 Chain	0.587	0.100	0.761	0.005
**ALPL**	Alkaline Phosphatase, Liver/Bone/Kidney	-0.228	0.147	-0.200	0.227
**BMP7**	Bone Morphogenetic Protein 7	-0.066	0.627	-0.014	0.852
**BMPR2**	Bone Morphogenetic Protein Receptor Type 2	-0.118	0.183	-0.428	0.047
**BMP2**	Bone Morphogenetic Protein 2	-0.031	0.824	0.339	0.039

Most of the genes had log Fold Change values >0.3 (> 2-fold ratio differential expression). The genes include those encoding for macromolecules, various components of NLRP3 inflammasome, and osteogenic proteins. All Microarray data have been deposited with Gene Expression Omnibus (GSE-89028).

Further we looked at the genes encoding for the macromolecules that have specific roles in modulating CaOx crystal formation and deposition in the hyperoxaluric rats. On comparing the relative gene expression of the control and the EG fed group, we found the results as anticipated for Osteopontin (OPN), Monocyte Chemoattractant Protein (MCP-1), Matrix Gla Protein (MGP), and Fetuin-B. For both Day 14 and Day 28, the genes encoding for the above said proteins were up-regulated and the relative level of up regulation was more for Day 28 as compared to Day 14. Similar results were found for the gene encoding for Lipocalin (Lcn2), Fibronectin (FN-1), Clusterin (CLU), CD44 and Alpha Microglobulin Bikunin precursor (AMBP) **(Table 6)**. Genes encoding for THP were however significantly downregulated on days, 14 as well as 28.

Major genes involved in the activation of the NLRP3 inflammasome were significantly up regulated on both day 14 and day 28 **(Table 6)**. These included PYCARD, NLRP3, IL-18, TXNIP, and IL-1β. Caspase-1 was non-significant on day 14 but was highly significant on day 28. Expression of the genes encoding for molecules involved in osteogenesis were also affected. Genes for Runt Related Transcription factor 1 (RUNX 1), Runt related Transcription factor 2 (RUNX 2), Cytokeratin -18 (KRT-18), Cytokeratin-8 (KRT-8), Vimentin (VIM), Collagen 1A1 (COL1A1), and Collagen 1A2 (COL 1A2) were up regulated on day 14 as well as day 28. Genes encoding for Alkaline Phosphatase (ALPL), Bone Morphogenetic Protein 7 (BMP 7), and Bone Morphogenetic Protein receptor 2 (BMPr2) were down regulated on both day 14 and day 28. Bone Morphogenetic Protein 2 (BMP 2) was down regulated on day 14 but was up regulated on Day 28 **(Table 6)**.

### Immunohistochemical staining

**Fig 4:**
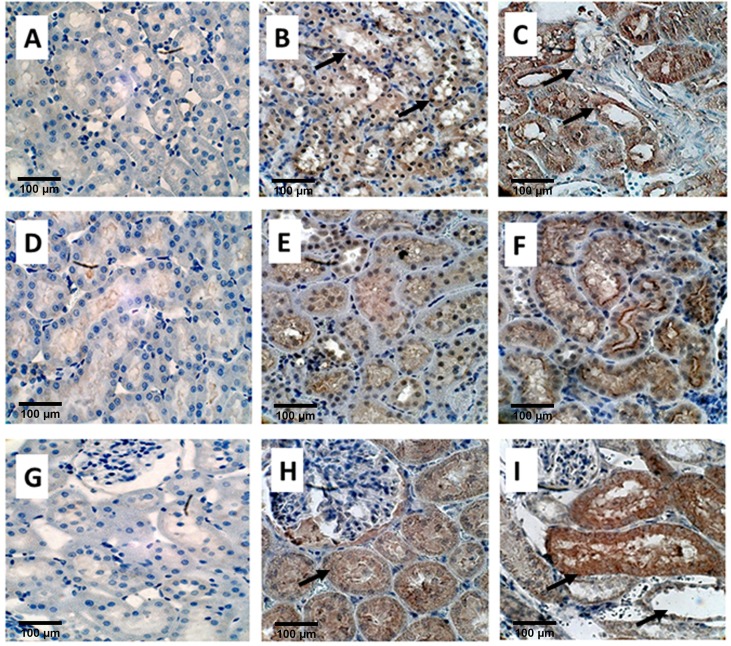
Immuno-histological staining of EG treated rat kidneys. **OPN**, A) Control B) on Day 14 showing light staining of the tubular epithelial cells, shown by black arrows and C) on Day 28 showing strong staining of renal tubular epithelial cells as well as tubular contents (Black arrows), Magnification, X 40, Scale bar 100μm, **MGP,** D) Control E) light staining on Day 14 and F) heavy staining on D28 Magnification, X 40, Scale bar 100μm. **Fetuin B**, G) Control, H) Non-specific diffuse staining of epithelial cells on Day 14, shown by a single black arrow, and I) Heavy staining of cells as well as luminal contents (top black arrow), particularly those with crystals which have dropped out during processing shown by bottom right black arrow.

**Fig 5:**
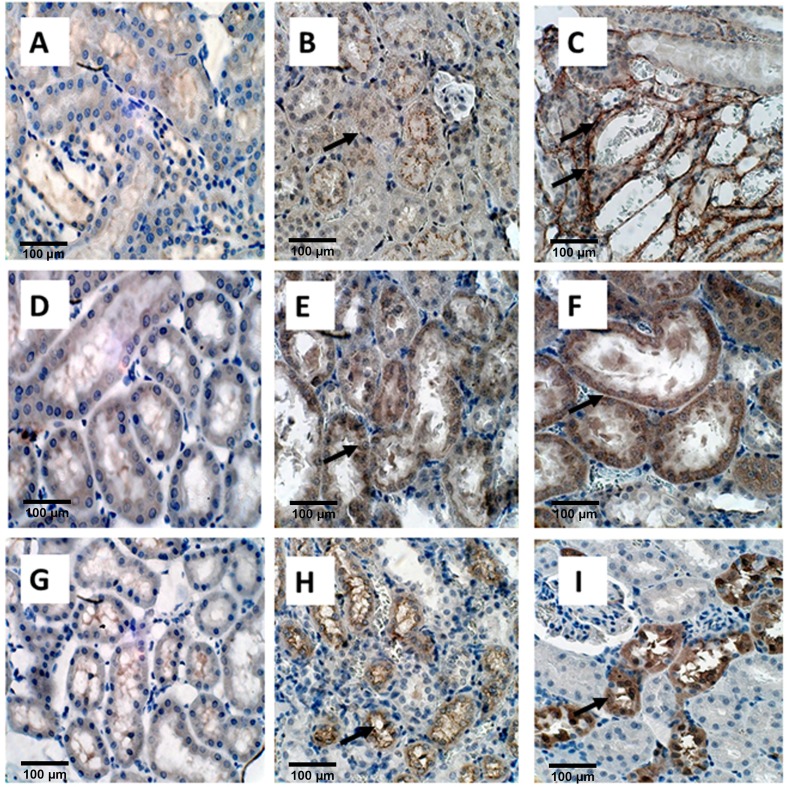
Immuno-histological staining of EG treated rat kidneys. **Collagen, A)** Control **B)** Light staining nonspecific staining on Day 14 shown by black arrow. **C)** Staining of the tubular basement membrane and interstitium on Day 28 shown by 2 black arrows. Magnification X40. **Fibronectin, D)** Control, **E)** strong staining of tubular epithelial cells on Day 14 (black arrow) and **F)** day 28 shown by black arrow. **THP, G)** Control **H)** Tubular epithelial cells stained positive on both Day 14 (black arrow) and **I)** Day 28, but number of positive cells appeared less on day 28 then on Day 14. Magnification X40, Scale bar 100 μm.

**Fig 6:**
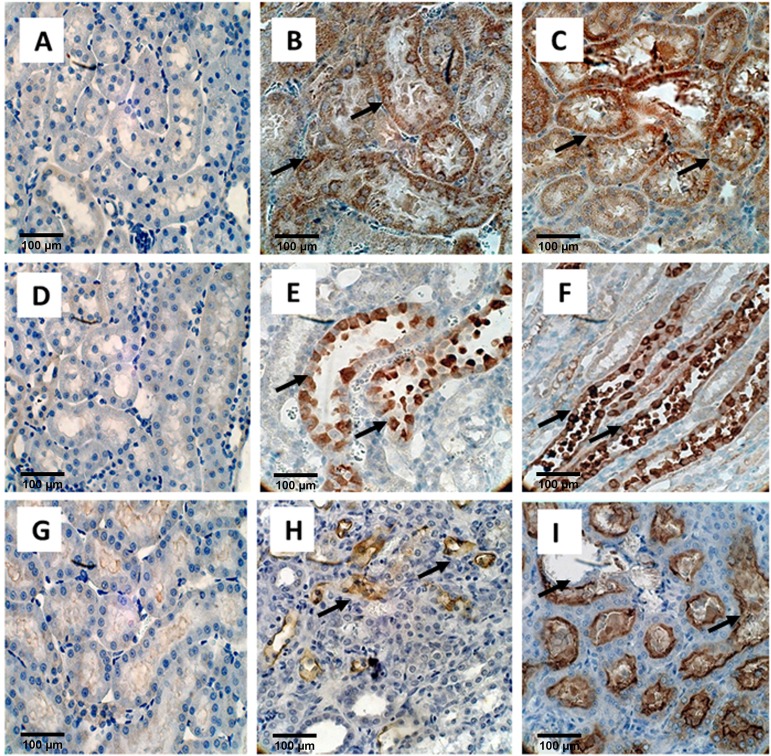
Immuno-histological staining of EG treated rat kidneys. **NOX2, A)** Control **B)** Day 14 **C)** Day 28. Staining is similar on both days and seen in both the renal tubular epithelial cells as well as interstitium shown by black arrows. NADPH oxidase 4 or **NOX4, D)** Control **E)** Day 14 and **F)** Day 28. Staining appears similar on both days and limited to the renal epithelial cells of the ascending limbs of the Loop of Henle (black arrows showing staining of renal epithelial cells). **KIM1, G) Control H)** Day 14 showing light localized staining (black arrows) and **I)** Day 28, showing large distended tubule from which crystals have fallen during processing (Black arrow) and there is heavy expression both in the tubular cells and lumens Magnification, X 40, Scale bar 100 μm.

Immunostaining was used to determine the expression of OPN, KIM-1, MGP, Collagen, FN1, THP, FETUB, NOX2, and NOX4 in the kidneys (**Figs 4, 5 and 6**). To some extant these molecules were expressed in the kidneys on both day 14 and day 28. However, the expression of most of these proteins was more pronounced on day 28 than on day 14 as compared to the control. Expression of OPN (**Fig 4A, 4B and 4C**), MGP (**Fig 4D, 4E and 4F**), and Fetuin B (**Fig 4G, 4H and 4I**), was much higher on day 28 than on day 14 as compared with control. As has previously been shown, heavy staining for OPN, MGP, and Fetuin on day 28 was seen, in association with the crystals, in the tubular lumens of distal tubules and collecting ducts). Light and nonspecific collagen staining in tubular epithelial cells was seen on day 14, whereas no staining was there in the control. On the other hand, heavy staining of tubular basement membrane and interstitium was observed after 28 days for Collagen as compared to the control (**Fig 5A, 5B and 5C**). Staining for Fibronectin appeared limited to the tubular epithelial cells. (**Fig 5D, 5E and 5F**) THP expression was obvious in the tubular epithelial cells as well as tubular lumens on both day 14 and 28. THP staining was however seen in fewer tubules on day 28 (**Fig 5G, 5H and 5I**).

Staining for various subunits of the NADPH oxidase was also affected by hyperoxaluria and the deposition of CaOx crystals in the kidneys. Nox 2 (**Fig 6A, 6B and 6C**) and Nox 4 (**Fig 6D, 6E and 6F**) appeared to increase in the cortical tubules, mostly in the cytoplasm of the collecting ducts, and distal tubules. Staining appeared more intense on day 28 than day 14 as compared to the control. Non- specific staining was also evident in the renal interstitium. Nox 4 staining was also seen in the ascending limbs of the loop of Henle. There was light localized staining on day 14 but strong staining with large distended tubules on day 28 for KIM1 and no staining for the control (**Fig 6E, 6F and 6G**).

## Discussion

CaOx kidney stone formation depends not only on the urinary environment but also how it shapes the renal response. Many animal model and tissue culture studies have been performed to understand this relationship between various urinary factors and renal epithelial cells, particularly the interaction between renal epithelium, oxalate and CaOx crystals [34–41]. Most studies looked for signs of injury, such as membrane permeability, cell viability, DNA synthesis, production of ROS etc. All of them agreed that CaOx crystals are injurious to renal epithelial cells. Studies of oxalate exposure however led to a range of conclusions. Oxalate toxicity was determined to be cell specific and time and concentration dependent. Collecting duct cells were found to be less susceptible to injury than cells of the proximal tubular origin. Genome wide analysis of differentially expressed genes in a line of proximal tubular epithelial cells, the HK-2 cells, in response to oxalate showed 750 upregulated and 2276 downregulated genes and 20 genes were differentially regulated irrespective of the duration of exposure [34].

Genome wide analysis of mice kidneys with hyperoxaluria induced by the administration of glyoxalate showed at least 2-fold increase in the expression of 25 genes during deposition of CaOx crystals. Gene ontology analysis showed increased expression of genes related to inflammation, immune reactions and complement activation pathways [42]. We analyzed changes in global transcriptome in the kidneys of rats given hydroxyl-L-proline for 4 weeks. Rats were hyperoxaluric and their kidneys showed heavy deposition of CaOx crystals. Analysis of 22,226 genes displayed 20 and 24 significant pathways in the cortex and medulla respectively. Genes encoding for PYCARD (ASC), TXNIP, NLRP3, caspase-1, and IL-1β and IL-18 were significantly upregulated [23]. Genes involved in the activation of NADPH oxidase were highly expressed with concurrent decrease in the expression of genes encoding for ROS scavenger proteins [31]. Expression of genes involved in the epithelial transformation and bone morphogenesis was also altered [28]. In addition, CaOx nephrolithiasis affected the expression of genes encoding for various crystallization modulators [27].

In the present study, we investigated differences in renal response to continuous exposure to high oxalate with or without the deposition of CaOx crystals by analyzing changes in global transcriptome of renal tissue, following EG induced hyperoxaluria in male rats. Emphasis was placed on the genes and pathways involved in the production of reactive oxygen species, crystallization modulators, development of inflammation and osteogenesis. We looked at the relative gene expression in hyperoxaluric and nephrolithic kidneys compared to the control group to determine the signaling pathways activated during hyperoxaluria and nephrolithiasis to better understand the pathophysiology of oxalate induced diseases of the kidneys. 15 pathways were common between hyperoxaluric and nephrolithic kidneys. Interesting thing to observe was that 18 unique pathways became significant only in nephrolithic kidneys while only 2 unique pathways were significant in the hyperoxaluric kidneys. Unique pathways in the nephrolithic rats included Nod-like receptor pathway which involves NFĸB signaling, MAPK signaling, apoptosis and activation of inflammasomes. Our previous study has already shown the involvement and activation of the Renin-angiotensin pathway along with DAG/PKC pathway of neutrophil activation [31].

Our study also considered important set of genes involved in the activation of NLRP3 (NACHT, LLR and PYD domains containing protein 3). We saw significant up regulation of genes encoding for PYCARD (PYD and CARD domain containing) also known as ASC (Apoptosis Associated Spec like protein), Caspase 1, IL-18 (Interleukin-18), TXNIP (Thioredoxin Interacting Protein), and IL-1β. Relative gene expression for Caspase-1 and IL-1β was, however, not significant at day 14 but when crystals were formed around day 28 we saw highly significant gene expression. Caspase-1 and IL-1β are important end products of NLRP3 inflammasome activation [43]. This indicates that inflammation pathways are activated during crystal formation as compared to hyperoxaluric conditions as shown in this study.

We further looked at genes encoding for proteins responsible for epithelial to osteogenic transformation including Runx-1 (Runt-related transcription factor-1), Runx-2, ALPL (Alkaline phosphatase), Bone morphogenetic proteins (BMP) genes such as BMP-2, BMP-7, BMPr2 as well as Cytokeratin’s such as KRT 8, KRT 18, Vimentin (VIM), Fibronectin (Fn-1), Collagen 1a (COL1a1) and COL1a2. Genes encoding for Runx1, Runx2, KRT 18, KRT 8, VIM, Fn-1, Col1a1 and Col1a2 were up regulated during hyperoxaluric conditions and further increased after crystal deposition or nephrolithiasis. Gene encoding for ALPL (Alkaline Phosphatase) was down regulated as shown in our previous study [28].

As mentioned earlier, CaOx nephrolithiasis in a rat model is associated with the activation of NADPH oxidase, production of ROS, development of oxidative stress and renal injury [22,44,45]. In our current study, Nox 2, Nox 4 as well as P22 were upregulated during hyperoxaluria as well as during nephrolithiasis suggesting early activation of NADPH oxidase. KIM-1, a recognized marker of renal injury [46], was highly expressed at both the gene and protein levels during nephrolithiasis. Glutathione Peroxidase (Gpx) is an antioxidant enzyme that is highly expressed in the kidney and removes peroxides and peroxynitrite that can cause renal damage. During oxidative stress, there is an increased expression of Gpx as shown previously. In our data, Gpx 2 was highly upregulated in the kidneys particularly during nephrolithiasis. Results indicate that CaOx crystals are more injurious to the kidneys.

Results also showed increased urinary excretion of LDH, a marker of membrane permeability and damage. The increase was evident early during hyperoxaluria indicating that oxalate alone can induce changes in the renal epithelial cells. Urinary creatinine levels were not affected during hyperoxaluria but increased after crystal deposition, highly significantly so on day 28 indicating renal damage.

As shown in previous studies [27,31] many macromolecular crystallization modulators are upregulated when renal epithelial cells are exposed to oxalate and CaOx crystals [24–27,47–49]. Results of our present study show that OPN, MGP, Fetuin B, Fn1, CD 44, Clusterin, Bikunin/AMBP genes are upregulated during hyperoxaluria and further increased with crystal deposition. Osteopontin is one of the most studied macromolecular modulator of CaOx and Calcium Phosphate (CaP) stone formation and plays a vital role in bio mineralization and crystallization of CaOx and CaP kidney stones [50–55]. Matrix Gla Protein (MGP) also known as the cell growth-inhibiting gene 36 protein is a vitamin K dependent protein whose main role is inhibitor of bone formation/vascular calcification [56]. Previous studies have shown increased expression of MGP in renal tubules of hyperoxaluric rats [57,58], as well as renal epithelial cell lines NRK-52E and MDCK on exposure to oxalate and CaOx crystals [58,59]. Fetuin-A and B are expressed in humans, rats and mice at both the mRNA and protein levels [60]. Both actively inhibit precipitation of basic calcium phosphate *in vitro*. Fetuin A is considered an important inhibitor of pathological calcification in the humans [61]. Fetuin A deficient mice develop soft tissue calcification, including nephrocalcinosis [62]. The role of fetuin in kidney stone formation has not been analytically examined; however, it has been reported that kidney stone patients have lower urinary Fetuin A levels than normal controls [63].

Clusterin, like Kim-1, is expressed on the tubular cells after kidney injury and is also induced in polycystic kidney disease [64], and renal cell carcinoma [65]. CD44 is a cell surface receptor of OPN and has been shown to be highly expressed during injury, inflammation and wound healing and plays a role in formation of peri cellular matrix on surface of proliferating and migrating cells. CD44 has been shown to be increased in the kidneys of hyperoxaluric rats [16]. The AMBP gene encodes for Bikunin and α1-microglobulin, both inhibitors of CaOx crystallization [66,67]. Previous studies have shown that expression of AMBP was increased in the renal tubular cells of hyperoxaluric rats [18,68]. Bikunin mRNA expression was increased when renal epithelial cells were exposed to oxalate and CaOx crystals [69]. Our microarray data also showed up regulation of AMBP gene in the hyperoxaluric rats. Fibronectin is a known inhibitor of CaOx kidney stones [70]. Previous studies based on microarray analysis of genes in renal epithelial cells exposed to CaOx crystals have shown high expression of Fibronectin [42,71,72]. Our study based on microarray analysis of genes obtained from the kidney of hyperoxaluric rat showed comparable results.

Studies have shown that renal epithelial cells on exposure to oxalate/CaOx crystals produce inflammatory molecules such as Monocyte Chemoattractant Protein (MCP-1) [73]. MCP-1 or Ccl2 (Chemokine -CC motif Ligand 2) recruits monocytes, T-cells and dendritic cells at site of renal injury/inflammation and plays a significant role in the progression of renal failure. Results of this study show that MCP-1 was only slightly increased during hyperoxaluria, but highly expressed in the kidneys with CaOx crystals indicating that crystal exposure plays a key role in its expression.

Lipocalin 2 (Lcn 2) also known as Neutrophil Gelatinase-Associated Lipocalin or NGAL is a critical inflammatory mediator, contributing to tubular damage and kidney failure [74]. In our data, Lcn2 was highly up regulated, indicating tubular damage due to CaOx crystals.

Interestingly THP gene was downregulated during hyperoxaluria as well as CaOx nephrolithiasis. Similar results were obtained in our previous study using hydroxyl-L-proline as a hyperoxaluria inducing agent [27], as well as another study where EG was used to induce hyperoxaluria [75]. Inactivation of THP gene increases the frequency and severity of CaOx crystal deposition during EG induced hyperoxaluria, compared to the wild type control mice. In addition absence of THP leads to OPN induction suggesting that THP serves as a constitutive inhibitor of crystallization while OPN may act as an inducible inhibitor [76].

In this study, we identified 15 pathways that were common between the hyperoxaluria (day 14) and nephrolithiasis (day 28) (**Table 3**). These common pathways do provide an up or down stream response to development of hyperoxaluria and subsequently CaOx crystal formation. Looking at the common pathways we see a number of immunological and pathological changes taking place in various cells of the renal tissue in response to an oxalate load. After the onset of hyperoxaluria, we see up regulation of different signaling pathways including the extracellular matrix, complement and coagulation cascades, cell adhesion molecules, focal adhesion and pathways in cancer, to name a few. We observe that the immune response of the animal is triggered as evident by the activation of signaling pathways such as chemokine signaling pathway, cytokine-cytokine receptor interaction, leukocyte transendothelial migration, natural killer cell mediated cytotoxicity, and Fc Gamma R-mediated phagocytosis. These pathways remain active and significantly expressed till crystal formation stage.

There were 18 unique pathways that were significantly expressed after or during the onset of crystals as shown in **Table 4**. These pathways include but are not limited to P53 signaling which can be triggered due to different stress signal and may result in cell cycle arrest and apoptosis, Nod-like receptor signaling which leads to the activation of various proinflammatory cytokines such as IL-18 and IL1-β, after the activation of various inflammasomes, Hypertrophic cardiomyopathy, dilated cardiomyopathy, Toll-like receptor signaling pathway, T-cell receptor signaling pathway and Renin-angiotensin system pathway. These unique pathways give us an insight into how to differentiate between the renal consequences of hyperoxaluria and nephrolithiasis.

## Conclusions

This transcriptional study of the ethylene glycol fed rat hyperoxaluric model at two different time intervals provides a better insight on the different pathways involved in hyperoxaluria and CaOx crystal deposition in the EG fed rat kidneys. Hyperoxaluria alone upregulates several genes, but crystal deposition further increases their expression. Hyperoxaluria is also associated with the activation of NADPH oxidase which generally leads to the production of ROS [44,77]. In addition crystal deposition is associated with substantially increased renal expression of KIM-1, and its urinary excretion [29], which is a well-recognized sign of renal injury [44]. Hyperoxaluria alone did not cause a change in urinary excretion of creatinine which was significantly increased after crystal deposition indicating renal damage. Apparently changes brought about by the exposure to oxalate without crystal deposition are not sufficient for noticeable renal injury. ROS produced during hyperoxaluria are most likely taken care of by antioxidant defense of the kidneys such as the upregulation of glutathione peroxidase as seen here.
